# The Influence of Fibrin Formation on the Transplantability of Murine Tumour Cells: Implications for the Mechanism of the Révész Effect

**DOI:** 10.1038/bjc.1974.68

**Published:** 1974-04

**Authors:** L. J. Peters, H. B. Hewitt

## Abstract

Experiments were undertaken to test a new hypothesis for the mechanism underlying the Révész effect. The hypothesis proposes that lethally irradiated (LI) tumour cells enhance the take probability of a small number of transplanted viable (V) tumour cells mixed with them by exerting a thromboplastic effect at the site of injection; local fibrin formation prevents emigration of V cells from the site or secures their survival there. The evidence presented to support this hypothesis is as follows: in the case of 3 isogeneically transplanted tumours, admixed particulate brain extract simulated the effect of LI cells in increasing the take probability of V cells; brain extract simulated the effect of LI cells in greatly delaying the disappearance of ^125^IUdR-labelled viable carcinoma cells from the injection site; V cells acquired a raised take probability by their incorporation in fibrin clots; it was confirmed that admixed erythrocytes increased the take probability of V cells; using a newly devised microscopical test for detection of the thromboplastic activity of individual cells, it was found that cell death was almost always required for the display of such activity; lymphocytes and bone marrow cells, ineffective in enhancing the take of V cells, were almost totally devoid of thromboplastic activity. Possible explanations are given for failure of a fibrinogen depleting agent, ancrod (Arvin) to inhibit the Révész effect when administered to recipients. It is concluded that the evidence strongly supports the hypothesis presented whilst seriously weakening the long-standing theories that admixed LI cells act by provision of nutrients or by local quenching of postulated immune reactivity.


Br. J. Cancer (1974) 29, 279

### THE INFLUENCE OF FIBRIN FORMATION ON THE TRANSPLANTABILITY OF MURINE TUMOUR CELLS: IMPLICATIONS FOR THE MECHANISM OF THE RÉVÉSZ EFFECT

L. J. PETERS AND H. B. HEWITT

*From the Gray Laboratory, Mount Vernon Hospital, Northwood, Middlesex HA6 2RN, England*

Received 3 January 1974. Accepted 16 January 1974

**Summary.—Experiments were undertaken to test a new hypothesis for the mechanism underlying the Révész effect. The hypothesis proposes that lethally irradiated (LI) tumour cells enhance the take probability of a small number of transplanted viable (V) tumour cells mixed with them by exerting a thromboplastic effect at the site of injection; local fibrin formation prevents emigration of V cells from the site or secures their survival there. The evidence presented to support this hypothesis is as follows: in the case of 3 isogeneically transplanted tumours, admixed particulate brain extract simulated the effect of LI cells in increasing the take probability of V cells; brain extract simulated the effect of LI cells in greatly delaying the disappearance of $^{125}$IUdR-labelled viable carcinoma cells from the injection site; V cells acquired a raised take probability by their incorporation in fibrin clots; it was confirmed that admixed erythrocytes increased the take probability of V cells; using a newly devised microscopical test for detection of the thromboplastic activity of individual cells, it was found that cell death was almost always required for the display of such activity; lymphocytes and bone marrow cells, ineffective in enhancing the take of V cells, were almost totally devoid of thromboplastic activity. Possible explanations are given for failure of a fibrinogen depleting agent, ancrod (Arvin) to inhibit the Révész effect when administered to recipients. It is concluded that the evidence strongly supports the hypothesis presented whilst seriously weakening the long-standing theories that admixed LI cells act by provision of nutrients or by local quenching of postulated immune reactivity.**

RÉVÉSZ (1956) first described the stimulation of the growth of transplanted murine tumours which results when a preponderance of lethally irradiated (LI) tumour cells are added to an inoculum of viable (V) cells. It is remarkable that further studies of this effect by Révész and by others during the subsequent 17 years have not led to an understanding of the mechanism of the effect.

In a recent report from this laboratory (Hewitt, Blake and Porter, 1973) the results of fully quantitative transplantation assays were described using a carcinoma ("N.T.") of spontaneous origin in a CBA/Ht mouse; the TD50 (number of viable cells required for successful transplantation to 50% of injected sites) was 7000 for viable cells injected alone and only 4 for viable cells injected in mixture with $10^5$ LI cells. (In the experiments reported here these values have changed to about 2100 and 11 respectively.) Under both conditions of transplantation, the relationship between the number of viable cells per inoculum and proportion of takes conformed to a Poisson distribution for single unit transplantation. From this and other evidence it was concluded that admixed LI cells acted by increasing the proportion of viable cells which contributed to tumour initiation, rather than by influencing the growth characteristics of the viable cells. Other findings of interest in the present context were as follows: unirradiated normal lymphocytes and LI normal marrow cells did not influence the TD50 for viable cells when injected in mixture with them; LI cells of other tumours, either of the same or of a foreign mouse strain, varied widely in their ability to reduce the TD50 when added to the inocula; LI cells of one allogeneic tumour (of the WHT/Ht mouse strain) reduced the TD50 to 25 cells; and LI cells of "N.T." itself were far more effective in reducing the TD50 than the use of whole-body irradiated mice for the assay of viable cells.

Overall consideration of the above findings, with special reference to the striking heterogeneity of the types of LI cell able to exert the effect, encouraged us to doubt the two most commonly asserted hypotheses for the mechanism of the Révész effect: that LI cells abrogate or saturate a low level of host immunity against the tumour cells and facilitate their survival and growth; or that the LI cells, or the products of their disintegration, restore a relative nutritional deficiency assumed to be suffered by the viable cells after their implantation into the tissues.

It has long been known that a variety of factors influence the ability of circulating tumour cells to implant and give rise to "metastatic" growth, whether the cells are injected intravenously or have been disseminated naturally from a primary implanted tumour (Wood, Holyoke and Yardley, 1961). Prominent among such factors have been blood coagulability, many investigators having found that anticoagulants reduce the tendency of disseminated cells to "seed" in the tissues. It occurred to us to explore possible analogies between the factors governing metastasis and those governing the ability of injected viable tumour cells to give rise to progressive tumours. The question arose whether LI cells enhance the conditions for growth of viable tumour cells injected subcutaneously by exerting a local thromboplastic influence. This paper reports a series of experiments by which an attempt was made to answer this question.

### MATERIALS AND METHODS

*Mice and tumours.*—Mice of 2 inbred strains were used: CBA/Ht and WHT/Ht. The tumours used were: CBA Carcinoma "N.T.", CBA Sarcoma "F", CBA Leukaemia IV, WHT Ascites Tumour I and WHT Squamous Carcinoma "G". All these tumours arose spontaneously and have been maintained by serial transplantation in the mouse substrain of origin. References to their use in previous experiments have been given by Hewitt *et al.* (1973).

*Transplantation assays of tumour cells.*—The methods for preparing single-cell suspensions from solid tumours have been described previously (Hewitt, 1966). The values of TD50 and their confidence limits were calculated from the data of assays by statistical methods described recently (Porter, Hewitt and Blake, 1973).

Figure 1 shows, for CBA Carcinoma "N.T.", the dependence of percentage tumour incidence on the log number of viable cells injected per site. The right hand curve is for viable cells injected alone, and the left hand curve is for viable cells injected in mixture with $3{\cdot}5 \times 10^4$ LI cells per inoculum. Comparison of these two curves shows that an inoculum of 200–300 viable cells (2·3–2·5 log) gives a take incidence of under 10% without LI cells, and an incidence approaching 100% with LI cells. The finding provides the basis of a number of our experiments using this tumour, in which the enhancing effect of an additive has been demonstrated by its ability to give a relatively high incidence of tumours from 200 to 300 viable cells.

*Preparation of LI tumour cells.*—Cell suspensions prepared as for viable cells were exposed to 7–8 krad while enclosed in a glass vial standing in an ice bath. All LI suspensions were tested for inactivation by injection into appropriate mice which were observed for at least 5 months; no suspensions so tested have given rise to tumours.

*Preparation of brain extract.*—Several brains were excised from normal mice and homogenized by grinding with sand. Phosphate buffered saline was added to give a final brain tissue concentration of 250 mg/ml. The diluted homogenate was lightly centrifuged to remove connective tissue fragments and sand and the opaque supernatant fluid was used in the experiments. In one experiment the supernatant was further centrifuged (2100 $g$ for 10 min) to provide an opalescent particle-free supernatant fluid.

*Preparation of clots as vehicles for tumour cell transplantation.*—Two techniques were used: *Preformed fibrin clots* were made by suspending viable tumour cells to the required density in a 10% solution of pure human fibrinogen (Koch–Light Laboratories) in saline; aliquots of 0·1 ml were clotted with thrombin *in vitro* and the discrete clots were implanted subcutaneously through a small incision. *In situ clots* were produced as follows: fresh mouse blood was collected into one-tenth its volume of 3·13% sodium citrate containing 10% $\epsilon$-amino caproic acid; platelet-poor plasma was separated by centrifugation and viable tumour cells were added to the required density; aliquots of 0·5 ml of the mixed suspension (to provide four 0·1 ml inocula per mouse) were recalcified immediately before injection; the 4 injections were completed within the 40–60 sec clotting time so that the inocula clotted *in vivo* almost immediately after injection; this was verified by the formation of palpable nodules at the injection sites.

*Experiments using cells labelled with 5-$^{125}$iodo-2$^{1}$-deoxyuridine ($^{125}$IUdR).*—The thymidine analogue $^{125}$IUdR is incorporated into the DNA of proliferating cells and is released only after cell death (Commerford, 1965). It is very little re-utilized (Dethlefson, 1971) and therefore provides a suitable label for studying the fate of injected tumour cells.

The method used for *in vitro* labelling of CBA " N.T." cells, based on that of Fidler (1970), was as follows: a tumour cell suspension was prepared as described above and approximately $4 \times 10^5$ viable cells were plated into each of twelve 5 cm plastic petri dishes with 4 ml of Eagle's MEM containing 15% foetal calf serum and $^{125}$IUdR 0·4 $\mu$Ci/ml (specific activity 1–6 mCi/mg). The cultures were incubated at 37°C in an atmosphere of air containing 5% $CO_2$. In some experiments, the $^{125}$IUdR was not added until the cells had been in culture overnight; in all cases, however, the cultures were harvested after an exposure of 24 hours to the labelled DNA precursor. After pouring off the medium, the cells were removed with phosphate buffered saline containing trypsin 0·2%, pancreatin 0·05%, and sodium citrate 0·3%; they were then washed repeatedly in phosphate buffered saline at pH 7·3 until the activity of the supernate per 0·1 ml was indistinguishable from background. The cells were finally re-suspended in Tyrode solution containing 5% mouse serum and were sedimented under gravity in narrow glass tubes to remove clumps. Counts of " viable " and " dead " cells were made on phase-contrast appearances. No evidence of radiotoxicity was observed with this technique, as measured by *in vivo* assays of cultured, labelled cells.

Labelling indices were determined by fixing a sample of the cells in methanol/glacial acetic acid (3 : 1) and exposing smears of these fixed cells to Ilford K5 nuclear liquid emulsion for 6 days. After developing, the autoradiographs were counterstained with aceto-orcein. The percentage of labelled cells so determined was at least 90% in these experiments.

All measurements of radioactivity were made with a LKB Wallac 80,000 automatic gamma sample counter; 5000 counts were accumulated for each sample. The level of activity attained was approximately 1 ct/sec/100 cells.

*Recognition of thromboplastic effect of individual cells.*—The following test was devised to permit comparison of thromboplastic activity between cells of different origins and morphology as seen by phase-contrast microscopy.

Pooled samples of human blood containing sodium EDTA as anticoagulant were centrifuged and the plasma was recovered. To 2 ml of EDTA treated plasma was added 5 i.u. of heparin followed by 2 mg $CaCl_2$, each in 0·1 ml of physiological saline. Suspensions of tumour or normal cells to be examined were well washed in Tyrode solution and diluted to contain approximately 3000 cells/mm$^3$. Equal volumes of cell suspension and prepared human plasma were mixed and the mixture was used to fill a Turck haemacytometer. The filled haemacytometer was incubated at 37°C for 20 min in a humidified chamber before examination by phase-contrast microscopy. A proportion of the cells in many such preparations display a very striking appearance, being surrounded by a corona of needle-like fibres extending radially from the cell margin; the length of the fibres extends gradually until they are several cell diameters in length. Further incubation is associated with increasing density of the fibres round affected cells, while initially unaffected cells usually remain quite free from fibres. Thus, differences between affected and unaffected cells appear to represent qualitative, rather than quantitative, differences of cellular thromboplastic capacity. Strong evidence that the fibres are of fibrin is provided by our observation that fibres do not form in serum, that they can be seen to disappear when trypsin is allowed to diffuse into the chamber, and that they form in great density around deposits of dried brain extract and around megakaryocytes. It is concluded that formation of the fibres is associated with coagulative factors accessible at the cell surface or secreted by the cells. For convenience, affected cells are referred to as " star " cells. The proportion of " star " cells in a suspension is determined by counting in relation to the density of living and dead cells present in the suspension.

*Treatment of mice with ancrod* (*Arvin*).—Arvin (Twyford Laboratories Ltd) is a purified enzyme fraction of Malayan pit viper venom. Injected into mammals, this has a thrombin-like action: fibrinogen is converted to fibrin which is removed by fibrinolysis, leaving the blood in a temporarily incoagulable condition from severe hypofibrinogenaemia. In the experiments reported by Hagmar (1972), repeated intraperitoneal injections of the drug into mice were complicated and restricted by the occurrence of intraperitoneal haemorrhage, presumably from damage to blood vessels by the injecting needle. We have completely avoided this complication by giving the injections in the mid-line through the avascular linea alba. Our schedule of treatment of CBA mice, based on Hagmar's detailed observations, was as follows: 3 doses of 50 u/kg body weight at 4-hour intervals, followed after 8 hours by 8-hourly injections of 100 u/kg; treatment was maintained for 4 days. Samples of tail vein blood taken from treated mice immediately before the administration of scheduled doses were taken into capillary tubes and incubated at 37°C; coagulation of blood was sought at intervals up to 16 hours by inspection of a capillary, and of its contents after their expulsion; no evidence of coagulation was seen in any specimen taken during the course of treatment. In view of Hagmar's (1972) detailed examination of the effect of ancrod on thrombin clotting time and fibrinogen concentration, we have not repeated these quantitative assessments of impaired blood coagulability. In the experiments to be reported, tumour cells were injected subcutaneously into ancrod-treated mice soon after injection of the third dose of 50 u/kg.

### EXPERIMENTS AND RESULTS

### 1. *Effect of admixed brain extract on the take of viable tumour cells*

Fully quantitated transplantation assays have been done with tumours CBA Carc. " N.T." and CBA Sarc. " F ", in which the effect of admixed brain extract, a rich source of thromboplastin, was compared with that of admixed LI cells of the corresponding tumours. The results of these assays are shown in Table I. The control assays for each intercomparison have been pooled. It is clear that the reduction of TD50 obtained with LI cells is closely approximated by brain extract.

In addition, single point assays of CBA " N.T." have been done to compare the efficacy of different preparations of brain extract: BE (1)—standard extract prepared as described previously in this paper; BE (2)—as above, except that no sand was used in grinding the brains; minimal sedimentation was used to remove any larger fragments of connective tissue but essentially this was a " whole " brain extract. BE (3)—the standard extract was centrifuged at 2100 *g* for 10 min to remove virtually all particulate material, leaving a " soluble " extract.

By using 200 V cells in these assays, excellent discrimination was obtained between effective and non-effective preparations (see Fig. 1). Results (tumour takes/sites injected) were as follows: BE (1), 23/24; BE (2), 24/24; BE (3), 1/28; controls (no brain extract), 4/28.

Thus, it is clear that the " soluble " extract is ineffective, whereas there is no difference between the standard and " whole " preparations. This difference, we believe, is due to the requirement of a sustained thromboplastic influence at the

TABLE I.—*TD50 Values for Viable Cells of Two Tumours Injected Alone, with LI Cells, or with Brain Extract (BE)*

| | TD50 (95% confidence limits) | | |
|---|---|---|---|
| Tumour | V only | V + LI | V + BE |
| CBA Carc. " N.T." | 2080 (1466–2951) | 11·1 (6·6–18·4) | 28·2 (17·1–46·6) |
| CBA Sarc. " F " | 611 (431–867) | 7·6 (4·6–12·7) | 8·9 (5·4–14·8) |

injection site; the particulate extracts fulfil this requirement while the soluble extract does not.

That the effect of brain extract is not peculiar either to the two tumours used, or to the CBA strain, was demonstrated by transplanting with or without brain extract 100 V cells of the WHT Sq. Ca. " G ". The tumour take incidences were, respectively, 22/24 and 4/24.

2. *Fate of $^{125}$IUdR-labelled tumour cells injected subcutaneously*

In these experiments it was necessary to use more than our usual 200–300 V cells in order to obtain measurable levels of activity per inoculum without radiotoxicity. The two experiments described here involved inocula of 800–1000 V cells, which, by reference to Fig. 1, still permit good discrimination between assays of V cells alone or with excess LI cells.

In the first experiment approximately 800 V cells of CBA " N.T." labelled with $^{125}$IUdR were injected subcutaneously, with or without 4 × $10^5$ LI cells, into the hind legs of mice. At intervals following injection, groups of mice were killed with ether, their legs were amputated and the residual activity at the injection sites was counted. Results are shown in Table II. It will be seen that by 5 days after injection, the residual activity was more than 30 times greater with added LI cells than without—a highly significant difference. It is reasonable to assume

FIG. 1.—Relationship between number of viable cells of CBA Ca. " N.T." per inoculum and tumour incidence. The open points are for viable cells injected alone (combined results of 2 assays) The solid points are for viable cells mixed with 3·5 × $10^4$ LI cells (results of one assay done in parallel with one of the above assays).

that the cells represented by this residual activity are those injected cells which actually contribute to tumour development, and it is of interest to compare the measured activity levels with the tumour take incidences which resulted from the same inocula as set out in Table III. Conversion of residual activity measurements to equivalent cells can be only approximate because of the contribution to initial activity of labelled non-viable cells and of non-DNA-bound label. Results of other experiments not reported here suggest that the rapid decline in activity over the first 12 hours after injection is due partly to these elements. Nonetheless, it is readily apparent that the effective "take unit" consists of only a very few V cells, and that at 120 hours the relative number of injected cells retained locally is greatly augmented by their admixture with LI cells; indeed, the "stimulation" of tumour growth can be interpreted in these terms.

In a similar experiment, the effect of brain extract in place of LI cells was observed. Figure 2 indicates graphically the qualitative similarity of the data, although with brain extract the final ratio of activities was 1 : 12 compared with 1 : 30 for LI cells. This is in accord with the slightly smaller effect of brain extract in reducing the TD50, as described previously in this paper.

### 3. *Evidence of retardation of cell emigration by brain extract*

When cells of CBA Leukaemia IV are injected subcutaneously, the recipient inevitably develops generalized leukaemia. In most cases no lesion develops at the injection site, though occasionally an ill-defined flattened infiltration may be noted.

If emigration of the leukaemia cells were retarded, two effects should be noted: local tumour growth and prolonged survival. In the present experiment, $10^4$ leukaemia cells were injected with or without brain extract into groups of 6–8 mice. After 17 days, when the

TABLE II.—*Residual $^{125}I$ Activity at Intervals after the Injection of* 800 *$^{125}IUdR$-labelled V Cells with or without Admixed LI Cells*

| Hours after injection | $^{125}$I activity (ct/sec ± s.e. mean)* V only | V + LI | t test |
|---|---|---|---|
| 0 | 13·01 ± 1·13 | | |
| 2 | 9·62 ± 2·04 | 12·72 ± 1·37 | $0·02 < P < 0·025$ |
| 6 | 4·47 ± 0·97 | 9·93 ± 3·11 | $P < 0·005$ |
| 19 | 0·77 ± 0·28 | 3·67 ± 0·65 | $P < 0·001$ |
| 50 | 0·083 ± 0·026 | 2·50 ± 0·49 | $P < 0·001$ |
| 120 | 0·046 ± 0·040 | 1·48 ± 0·34 | $P < 0·001$ |

* 6 sites per group; measurements corrected for background.

TABLE III.—*Correlation of Residual $^{125}I$ Activity Measurements with Tumour Take Incidence from Same Inocula*

| | 800 V cells only | 800 V cells + (4 × $10^5$) LI cells |
|---|---|---|
| Actual take incidence | 1/14 | 16/16 |
| Expected take incidence (from Fig. 1) | 1–3/14 | 16/16 |
| Residual activity at 120 hours: ct/sec for individual sites | 0·000, 0·000, 0·024<br>0·052, 0·089, 0·114 | 1·10, 1·18, 1·34<br>1·48, 1·87, 1·89 |
| "Equivalent" V cell number* | 0, 0, 1·5, 3, 5·5, 7 | 68, 73, 82, 91, 115, 116 |

* See text.

Fig. 2.—Rate of loss of viable cells of CBA " N.T." from subcutaneous injection sites, as measured by residual $^{125}$I activity following prior incorporation of $^{125}$IUdR into the DNA of the cells. Graph (a) indicates the activity loss when 800 viable cells were injected alone (solid points) or mixed with $4 \times 10^5$ lethally irradiated cells (open points). Graph (b) shows the corresponding curves for 1000 viable cells injected alone (solid points) or mixed with brain extract (open points). Error bars represent the s.e. mean of 6 sites for each data point.

disease was well developed, the mice were killed, the presence or absence of a local tumour was noted and the spleens were weighed. Results are shown in Table IV. The increase of local tumour incidence with brain extract is not statistically significant, but the reduction in spleen weights is highly significant ($P < 0{\cdot}005$). Brain extract injected remotely had no effect on evolution of the disease.

TABLE IV.—*Effect of Brain Extract (BE) on Dissemination of Leukaemia Cells from Subcutaneous Sites of Injection*

| Inoculum | Local tumour incidence | Mean weight of spleen (g) ± s.e. |
|---|---|---|
| $10^4$ Leukaemia cells | 1/6 | 1·128±0·101 |
| $10^4$ Leukaemia cells + admixed BE | 5/8 | 0·681±0·277 |
| $10^4$ Leukaemia cells + BE at remote site | 1/8 | 1·172±0·284 |

These results clearly suggest an inhibiting effect of admixed brain extract on the dissemination of leukaemia cells from the site of injection. The alternative explanation, that the brain extract was toxic to the cells, is made untenable by our observation that brain extract increased the take probability from limited numbers of viable cells of the three solid tumours tested.

4. *Transplantation of tumour cells in fibrin clots*

The demonstration that brain extract greatly reduced the number of V cells required for transplantation in at least 3 different systems suggested that the formation of a fibrin mesh at the injection site might be an important factor in the establishment of a successful graft.

In the first experimental design, we observed the result of implanting 300 V cells of CBA "N.T." in *preformed fibrin clots*. Progressive tumours developed in all recipients (6/6); the probability of this occurring with 300 V cells injected alone is less than 1 : 1000 (see Fig. 1).

To exclude the possibility that surgical trauma associated with surgical implantation influenced the take probability, we assayed 210 V cells of CBA "N.T." in *in situ clots* by the technique described in the previous section. The resulting tumour incidence was 20/20.

Thus, it is apparent that the number of cells required for tumour take was reduced dramatically by incorporation of cells in clots by both techniques.

The mean latent period before appearance of tumours from preformed fibrin clots (40 days) was rather long (Hewitt *et al.*, 1973), possibly reflecting the increased diffusion distances required for provision of cell nutrients. Successful tumour takes with relatively small numbers of V cells under conditions which increase the diffusion distances for nutritional provision argue strongly against the assertion that nutritional deficiences limit the take of small numbers of cells injected directly into the subcutaneous tissue without additives.

5. *Effect of added erythrocytes on tumour take incidence*

The finding of Yatvin, Stone and Clifton (1973) that growth of the MTGB carcinoma was promoted by the addition of washed erythrocytes to the tumour cell inoculum prompted us to test this adjuvant in a transplantation assay using CBA "N.T." Freshly collected citrated mouse blood was centrifuged and the packed cells were washed twice in ice-cold saline. A 33% suspension by volume of these cells in Tyrode solution was then used as a vehicle for assay of 280 V cells of the CBA "N.T." The tumour take incidence was 12/24, compared with 0/24 in a parallel assay of 280 V cells alone.

The results confirm the finding of Yatvin *et al.* (1973), using a different tumour and end point. It is relevant to note here that erythrocyte lysate has thromboplastic (Quick, Georgatsos and Hussey, 1954) and antifibrinolytic (Blofield and Hawkey, 1967) properties.

### 6. *Thromboplastic activity of individual cells*

Using the microscopic technique described in the previous section for recognition of thromboplastic activity by individual cells (" star " cells), we have examined cell preparations from 4 different normal tissues and 16 different tumours of the mouse. The results of these examinations are shown in Table V. Cell suspensions prepared by mechanical or enzymic disintegration of tissues are widely variable in their content of cells which are dead by morphological criteria, and it was noted that formation of " stars " was restricted almost exclusively to dead cells. Table V indicates for each suspension examined, whether significant numbers of dead cells or of " star " cells were seen; the percentage of *dead cells* presenting as " star " cells was determined for half the preparations. The Table shows that absence of " star " cells was sometimes associated with a scarcity of dead cells; on the other hand, suspensions containing numerous dead cells were quite variable in their content of " star " cells. In general, lymphoid tissues yielded few or no " star " cells, whereas solid sarcomata or carcinomata often yielded many. Cells of the ascites tumour could not be converted to " star " cells by killing them by heat, or with dilute alcohol from which the cells were washed free before testing.

Our observation that cell death almost invariably precedes the exhibition of pericellular thromboplasia suggests that admixed cells which are able to promote the initiation of tumours from small inocula of viable cells may actually require preliminary lethal irradiation as a condition for expression of their thromboplastic activity.

### 7. *Failure of ancrod to inhibit the effect of LI cells*

If the enhancement of tumour takes by admixed LI cells is associated with a thromboplastic effect of the latter, their influence should be reduced or abolished using recipients having a defect of blood coagulation which precluded fibrin formation.

A mixture of 200 V and 225,000 LI cells of CBA " N.T." was injected subcutaneously in 4 sites in each of 10 CBA mice in which a severe blood coagulation

TABLE V.—*Thromboplastic Activity of Individual Cells in Suspensions Prepared from Various Normal and Malignant Tissues*

| Tissue or Tumour | Presence of dead cells | " Star " cells | % dead cells forming " stars " |
|---|---|---|---|
| Nodal lymphocytes | ± | — | .. |
| Spleen cells | ± | — | .. |
| Marrow cells | — | ±* | .. |
| Peritoneal cells | + | ± | 1·6 |
| WHT lymphosarcoma I | + | + | .. |
| WHT lymphosarcoma II | + | + | .. |
| WHT ascites tumour | + | — | < 1 |
| CBA lymphoma | + | ± | < 1 |
| CBA ascites leukaemia | + | ± | < 1 |
| CBA lymphosarcoma | + | — | < 1 |
| CBA thymoma | ± | — | .. |
| WHT bone sarcoma I | + | + | .. |
| WHT bone sarcoma II | + | + | 11 |
| CBA sarcoma " S " | + | + | 64 |
| CBA fibrosarcoma | + | + | 4 |
| CBA chondrosarcoma | + | — | .. |
| WHT Sq. Ca. " D " | + | + | 30 |
| WHT Sq. Ca. " G " | + | + | 34 |
| CBA Carc. " N.T." | + | + | 20 |
| WHT Carc. " M.T." | + | + | .. |

* The only two " star " cells seen in the marrow preparation were both megakaryocytes.

defect had been induced by repeated injections of ancrod according to the schedule of treatment described in the previous section. Ten control mice received the same inocula of tumour cells but were given injections of saline at the same times as the test mice received ancrod. Although the blood of the ancrod treated mice was shown to be incoagulable from some hours before until 4 days after injection of tumour cells, the final incidences of tumours and the mean latent periods before their appearance (16·5 days) were not significantly different between the two groups: 40/40 sites developed tumours in the treated group, and 38/40 in the control group.

The results of this experiment do not support the hypothesis it was designed to test. On the other hand, it is conceivable that blood incoagulability was not maintained for a sufficiently long period. Further consideration of this experiment appears in the Discussion.

### DISCUSSION

An association between the growth or metastasizing potential of tumours and the formation of fibrin has been the subject of many publications during the last two decades. O'Meara (1958) suggested that growth of a tumour is conditional upon the prior formation of a fibrin lattice in the adjacent tumour bed and that this formation is maintained by the tumour itself. Many investigators have studied the effect of perturbations of the blood coagulation system of animals on the metastasizing potential of tumour cells embolized from tumours or injected intravenously. It is not our purpose to review the extensive reports on this topic, which are by no means unanimous in respect of their findings and interpretations. However, it may be said that the balance of evidence favours the view that tumour growth or the seeding of embolized cells is enhanced by conditions which encourage the formation or stability of fibrin and is decreased by treatments which inhibit coagulation or encourage fibrinolysis (*e.g.* Ketcham *et al.*, 1971; Cliffton and Agostino, 1964).

Consideration of the above suggested to us that the promotion of tumour take incidence by admixed LI tumour cells might be associated with a thromboplastic effect of such cells. Strong evidence in favour of this interpretation was provided by our demonstration that brain extract, a rich source of thromboplastin, simulated the effect of LI cells in all of the 3 tumour systems examined. The similar but less powerful effect of admixed erythrocytes, first demonstrated by Yatvin *et al.* (1973), provided further supportive evidence, in that Quick *et al.* (1954) have shown that erythrocyte lysate is thromboplastic. The fact that incorporation of viable cells in pure fibrin clots before implantation enhanced their capacity to give rise to tumours is very cogent evidence that stimulation of local fibrin deposition may be implicated in the Révész effect.

In a previous publication from this laboratory (Hewitt *et al.*, 1973) evidence was presented to show that the effect of admixed LI cells was to increase the number of injected clonogenic cells contributing to initiation of a tumour. The result of such increase would be to raise the take incidence from a given number of viable cells and to reduce both the latent period before appearance of tumours and the time taken for tumours to reach a specified, or lethal, size. A possible interpretation is that many of the viable cells injected without additive are lost from the site of injection, the effect of the additive being to retain them there. The results of our experiments with $^{125}$IUdR-labelled viable cells support this interpretation and serve to confirm the analogy between brain extract and LI cells. These experiments do not in themselves inform us whether viable cells lost from the injection site die on site or only after their emigration from it. That they *are* destroyed is proved by their failure to give rise to tumours elsewhere in the animal. It is pertinent here to refer to our experience that TD50 values for intravenous injection are commonly much higher than those for subcutaneous injection; in the case of the CBA Carcinoma "N.T." studied by us, the respective values were $> 67,000$ and about 2000 cells; similar findings for two other tumours are reported by Boeryd, Lundin and Norrby (1971). It is clear that cells which remain in a subcutaneous injection site have a relatively high chance of survival and replication and that those which leave are exposed to a high risk of destruction by unidentified mechanisms.

In assessing the thromboplastic activity of nucleated normal or malignant tissue cells, we have been very largely guided by our microscopic technique for observing the thromboplastic activity of individual cells. It is significant that lymphocytes and bone marrow cells gave very little evidence of thromboplastic activity by this test; it was reported previously that both these cell types were entirely without effect on the TD50 when mixed with viable cells of CBA Carcinoma "N.T." (Hewitt *et al.*, 1973). Our most important observation using the test was that thromboplastic activity was almost totally confined to cells whose morphological appearance indicated that they were dead, a significant exception being megakaryocytes seen in the marrow cell preparation. Moreover, the dead cells of different cell suspensions varied widely in their thromboplastic activity. This distinction between the thromboplastic exertions of living and dead cells clearly cannot be made using tissue extracts tested by macroscopic techniques. Our observation suggests that lethal irradiation of added cells may serve to ensure a sustained release of thromboplastic material at the site of injection over the period required. Cell killing by radiation is distinguished from most other methods of cell killing in that the time between exposure to the noxious influence and cell death varies very widely among individual cells; thus, an input of dead cells into the injection site would be sustained for several days. It is of interest in this context to note that large numbers of cells are known to die within established tumours (see Steel, 1968). It can be conjectured that progressive growth of a solid tumour, as well as its initiation, may sometimes be conditional upon influences which these dead cells exert.

In view of the large measure of support for our hypothesis, our failure to demonstrate inhibition of the Révész effect by treatment of injected mice with the defibrinating agent, ancrod, deserves further consideration. As judged by the rate of loss of $^{125}$IUdR-labelled cells, ancrod treatment was continued sufficiently long *after* injection of tumour cells; however, it is possible that treatment was not started sufficiently long *before* injection of tumour cells to deplete fibrinogen in the interstitial tissue. Alternatively, fibrin deposited by the action of ancrod itself may have persisted long enough to provide a suitable micro-environment for tumour initiation. We propose to undertake further studies with ancrod.

Our finding that pure fibrin clots and erythrocytes, both devoid of nuclear material, have some capacity to simulate the effect of LI cells is discouraging to the theory that LI cells act by providing essential nutrients not otherwise available to the injected tumour cells. Evidence and arguments against a rival theory—that LI cells act by inducing local immunosuppression—have been presented elsewhere (Hewitt *et al.*, 1973).

We have considered the possibility that the thromboplastic influence we have consistently found for effective additives has some equivalence with the tumour angiogenesis factor described by Folkman *et al.* (1971). Whilst the two factors could conceivably be elaborated by a single additive, their distinction is suggested by our observation that brain extract, a potent simulator of LI tumour cells, had no angiogenic effect when tested by the implantation of millipore chambers containing it.

Our present understanding of the mechanism of the Révész effect can be summarized as follows: it is suggested that, in the case of tumours having a relatively high TD50 by the subcutaneous route, a large proportion of viable cells injected alone either die on site or after their emigration from the site; the addition of LI cells (and of some other additives) to the inocula ensures a sustained thromboplastic influence at the site with the production of a fibrin lattice which either prevents emigration of viable cells or secures their survival at the site. The fibrin lattice may also provide the conditions required for stromification of the growing tumour, as suggested by O'Meara (1958). We suggest that tissue cell additives do not release an effective level of thromboplastic activity unless and until they undergo degenerative changes.

The superiority of some LI tumour cell suspensions in exerting the Révész effect may be attributable to a high content of thromboplastic agents in tumour cells; it may also be partly dependent on the high proportion of cycling cells in such suspensions, for an attempt at mitosis is usually required for the expression of radiation damage by cell death.

The TD50 for viable tumour cells is commonly much reduced when the mice used for transplantation assay have received sublethal whole-body irradiation (WBI). An almost unchallenged interpretation of this finding is that WBI suppresses an effective level of immunity in the recipients. However, since the phenomenon is commonly observed using isogeneic tumour host systems in which no other evidence of transplantation immunity can be demonstrated, we have been encouraged to question whether suppression of immunity is implicated in this effect. It is known that sublethal WBI, in rats at least, stimulates a substantial increase in the rate of synthesis of fibrinogen (John and Miller, 1968) with peaks at 3 hours and 4 days after exposure (Nadkarni and Samuel, 1973). Our present investigations are directed to an examination of possible common factors linking the mechanisms whereby locally injected LI cells, and WBI of the recipients, reduce the TD50 values for viable cells of solid tumours transplanted within isogeneic tumour–host systems.

The question arises whether the Révész effect, exerted by the mechanism we have suggested, has implications for the course or response to therapy of clinical cancer. We have already referred to a possible analogy between the conditions influencing seeding of metastases from embolized cells and those affecting the initiation of tumours from small numbers of transplanted viable tumour cells. A further consideration is whether the stimulation of growth of surviving cells which has been demonstrated in irradiated tumours undergoing regression (van Peperzeel, 1972) is partly dependent on a local release of thromboplastic substances from non-surviving cells during expression of their radiation damage. Finally, we suggest a possible relevance of our studies to the uncommon but well-documented phenomenon in which clinical recurrences or metastases coincide geometrically with an irradiated area. It seems to us that release of tissue thromboplastins associated with radiation damage could underlie this phenomenon, in the same way as such release is invoked to explain the predisposition of sites of accidental or surgical trauma to tumour recurrence or metastasis. This understanding appears preferable to that provided by the widely current and unsupported hypothesis that irradiation suppresses locally a form of immunity not independently demonstrable. The interpretation we have suggested allows us to reconcile the apparent contradiction between experiments which demonstrate increased tumour "takes" in acutely irradiated lung (van den Brenk *et al.*, 1973) and those which have shown a reduced rate of growth of tumours transplanted to pre-irradiated sites (Stenstrom *et al.*, 1955; Hewitt and Blake, 1968).

We are grateful to Miss Eileen Blake for her skilled technical assistance in all the experiments, to Miss Angela Walder for the breeding and care of all the mice used and for assistance in monitoring the tumour transplant systems, and to Dr N. J. McNally for help and advice in the labelling of cells *in vitro*. We are indebted to Dr E. H. Porter for many helpful discussions and suggestions. We gratefully acknowledge a generous gift of Arvin from Twyford Laboratories Ltd. The cost of the research was met exclusively by the Cancer Research Campaign.

### REFERENCES

BLOFIELD, A. & HAWKEY, C. (1967) Intravascular Haemolysis and Coagulation. *Lancet*, i, 852.

BOERYD, B., LUNDIN, P. M. & NORRBY, K. (1971) Tumour Growth after Intravenous, Intraperitoneal and Subcutaneous Injection of Syngeneic Monodispersed Tumour-Cell Suspension. *Eur. J. Cancer*, **7**, 557.

CLIFFTON, E. E. & AGOSTINO, D. (1964) Effect of Inhibitors of Fibrinolytic Enzymes on Development of Pulmonary Metastases. *J. natn. Cancer Inst.*, **33**, 753.

COMMERFORD, S. L. (1965) Biological Stability of 5-iodo-2′-deoxyuridine Labelled with Iodine-125 after its Incorporation into the Deoxyribonucleic Acid of the Mouse. *Nature, Lond.*, **206**, 949.

DETHLEFSON, L. A. (1971) An Evaluation of Radioiodine-labelled 5-iodo-2′-deoxyuridine as a Tracer for Measuring Cell Loss from Solid Tumor. *Cell & Tiss. Kinet.*, **4**, 123.

FIDLER, I. J. (1970) Metastasis: Quantitative Analysis of Distribution and Fate of Tumor Emboli Labelled with $^{125}$I-5-iodo-2′-deoxyuridine. *J. natn. Cancer Inst.*, **45**, 773.

FOLKMAN, J., MERLER, E., ABERNATHY, C. & WILLIAMS, G. (1971) Isolation of a Tumor Factor Responsible for Angiogenesis. *J. exp. Med.*, **133**, 275.

HAGMAR, B. (1972) Defibrination and Metastasis Formation: Effects of Arvin on Experimental Metastases in Mice. *Eur. J. Cancer*, **8**, 17.

HEWITT, H. B. (1966) The Effect on Cell Survival of Inhalation of Oxygen under High Pressure during Irradiation *in vivo* of a Solid Mouse Sarcoma. *Br. J. Radiol.*, **39**, 19.

HEWITT, H. B. & BLAKE, E. R. (1968) The Growth of Transplanted Murine Tumours in Pre-irradiated Sites. *Br. J. Cancer*, **22**, 808.

HEWITT, H. B., BLAKE, E. R. & PORTER, E. H. (1973) The Effect of Lethally Irradiated Cells on the Transplantability of Murine Tumours. *Br. J. Cancer*, **28**, 123.

JOHN, D. W. & MILLER, L. L. (1968) Effect of Whole Body X-irradiation of Rats on Net Synthesis of Albumin, Fibrinogen, $\alpha_1$–Acid Glycoprotein, and $\alpha_2$–Globulin (Acute Phase Globulin) by the Isolated, Perfused Rat Liver. *J. biol. Chem.*, **243**, 268.

KETCHAM, A. S., SUGARMAKER, E. V., RYAN, J. J. & ORME, S. K. (1971) Clotting Factors and Metastasis Formation. *Am. J. Roentg.*, **111**, 42.

NADKARNI, G. D. & SAMUEL, A. M. (1973) Effect of Whole-body X-irradiation on Plasma Protein Synthesis: Role of Adrenals. *Int. J. Radiat. Biol.*, **23**, 469.

O'MEARA, R. A. Q. (1958) Coagulative Properties of Cancers. *Ir. J. med. Sci.*, **394**, 474.

PORTER, E. H., HEWITT, H. B. & BLAKE, E. R. (1973) The Transplantation Kinetics of Tumour Cells. *Br. J. Cancer*, **27**, 55.

QUICK, A. J., GEORGATSOS, J. G. & HUSSEY, C. V. (1954) Clotting Activity of Human Erythrocytes: Theoretical and Clinical Implications. *Am. J. med. Sci.*, **228**, 207.

RÉVÉSZ, L. (1956) Effect of Tumour Cells Killed by X-rays upon the Growth of Admixed Viable Cells. *Nature, Lond.*, **178**, 1391.

STEEL, G. G. (1968) Cell Loss from Experimental Tumours. *Cell & Tiss. Kinet.*, **1**, 193.

STENSTROM, K. W., VERMUND, H., MOSSER, D. G. & MARVIN, J. F. (1955) Effect of Roentgen Irradiation on the Tumour Bed. I. The Inhibiting Action of Local Pretransplantation Roentgen Irradiation (1500 R) on the Growth of Mouse Mammary Carcinoma. *Radiat. Res.*, **2**, 180.

VAN DEN BRENK, H. A. S., BURCH, W. M., ORTON, C. & SHARPINGTON, C. (1973) Stimulation of Clonogenic Growth of Tumour Cells and Metastasis in the Lungs by Local X-irradiation. *Br. J. Cancer*, **27**, 291.

VAN PEPERZEEL, H. A. (1972) Effects of Single Doses of Radiation on Lung Metastases in Man and Experimental Animals. *Eur. J. Cancer*, **8**, 665.

WOOD, S., HOLYOKE, E. D. & YARDLEY, J. H. (1961) Mechanisms of Metastasis Production by Blood-borne Cancer Cells. *Can. Cancer Conf.*, **4**, 167.

YATVIN, M. B., STONE, H. B. & CLIFTON, K. H. (1973) Tumor Growth Stimulation in Mice by Radiation Killed Tumour Cells and Erythrocytes. In *Vth International Symposium on Cancer*. Ed. L. Severi. Perugia: University of Perugia Publications, 1973.


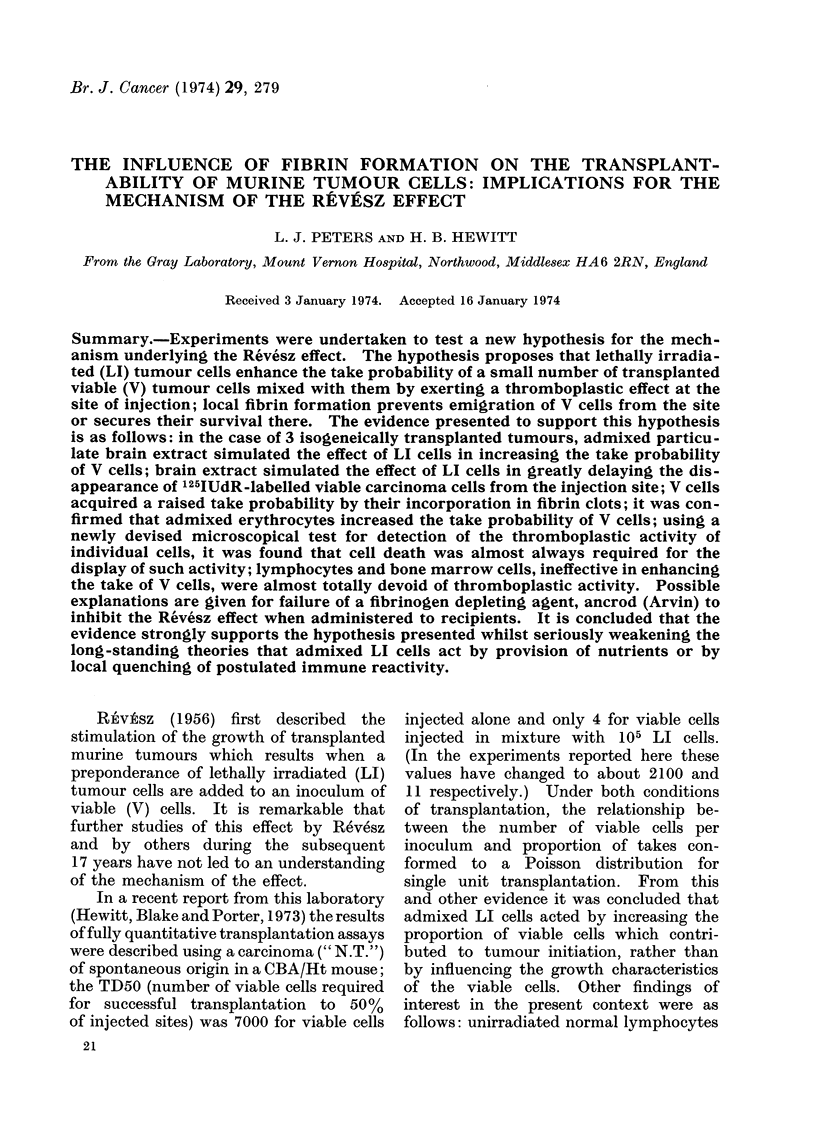

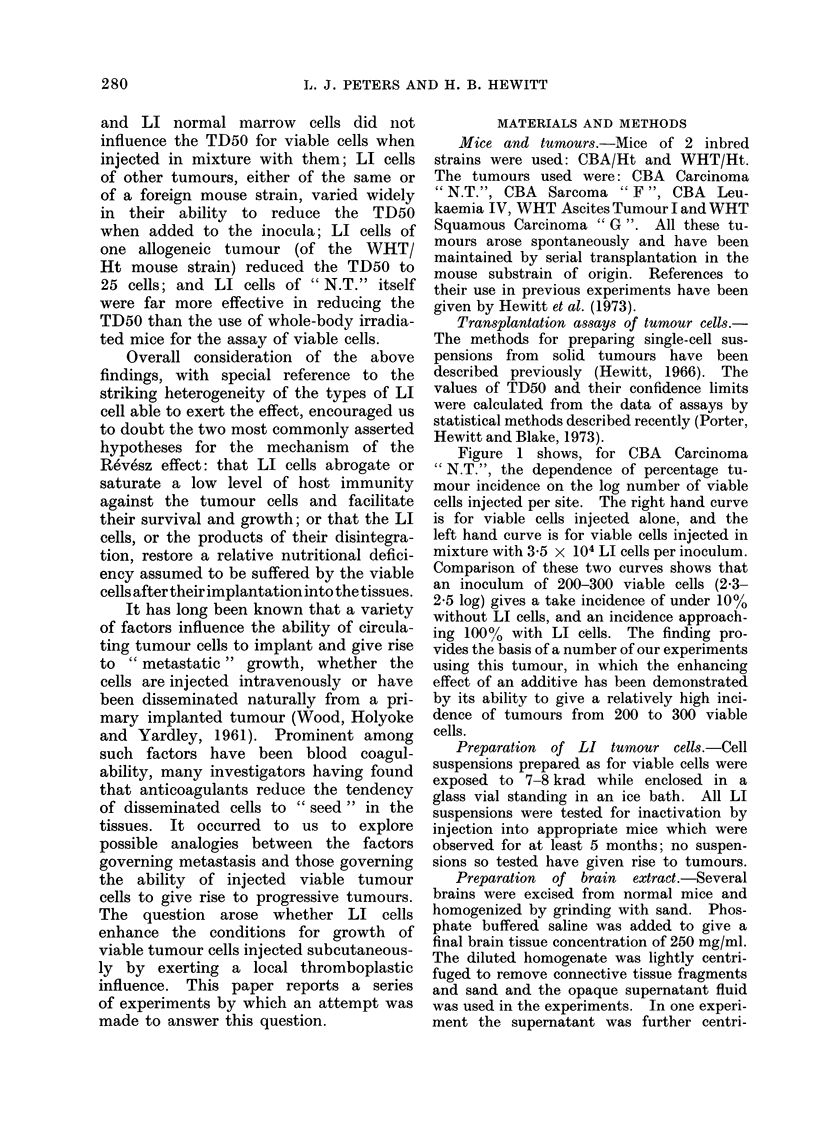

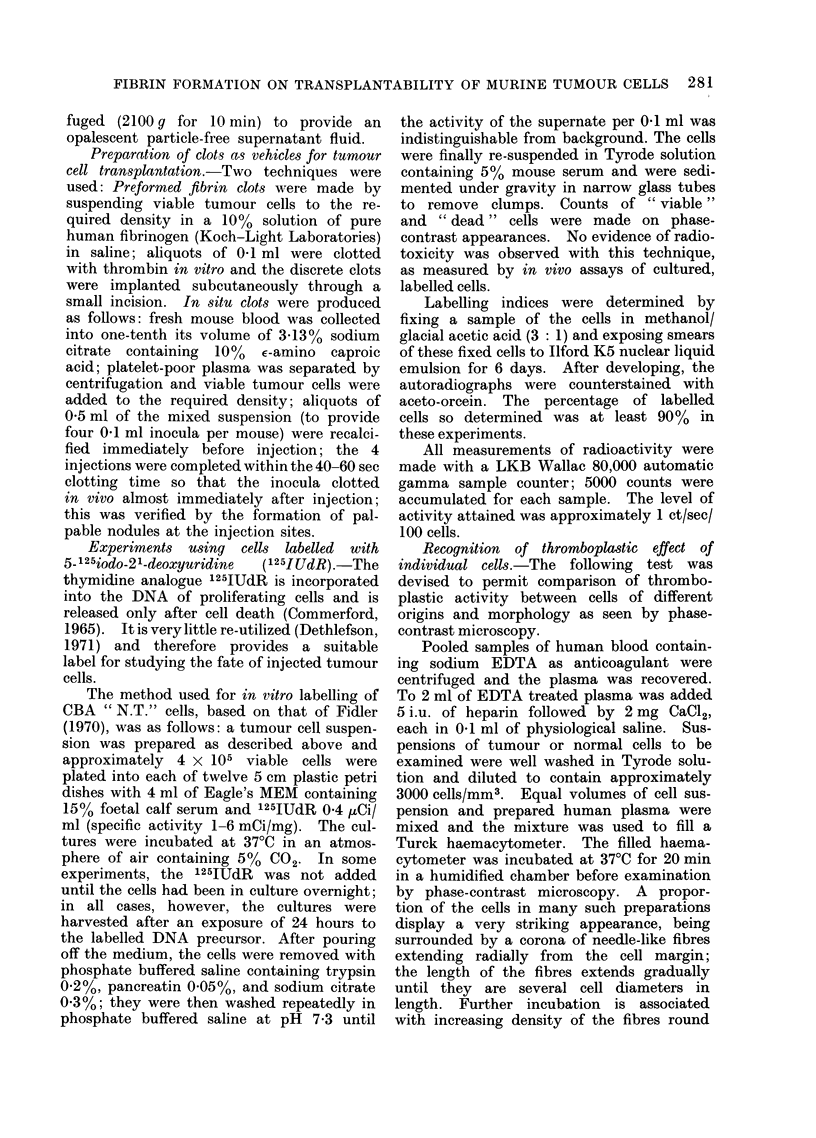

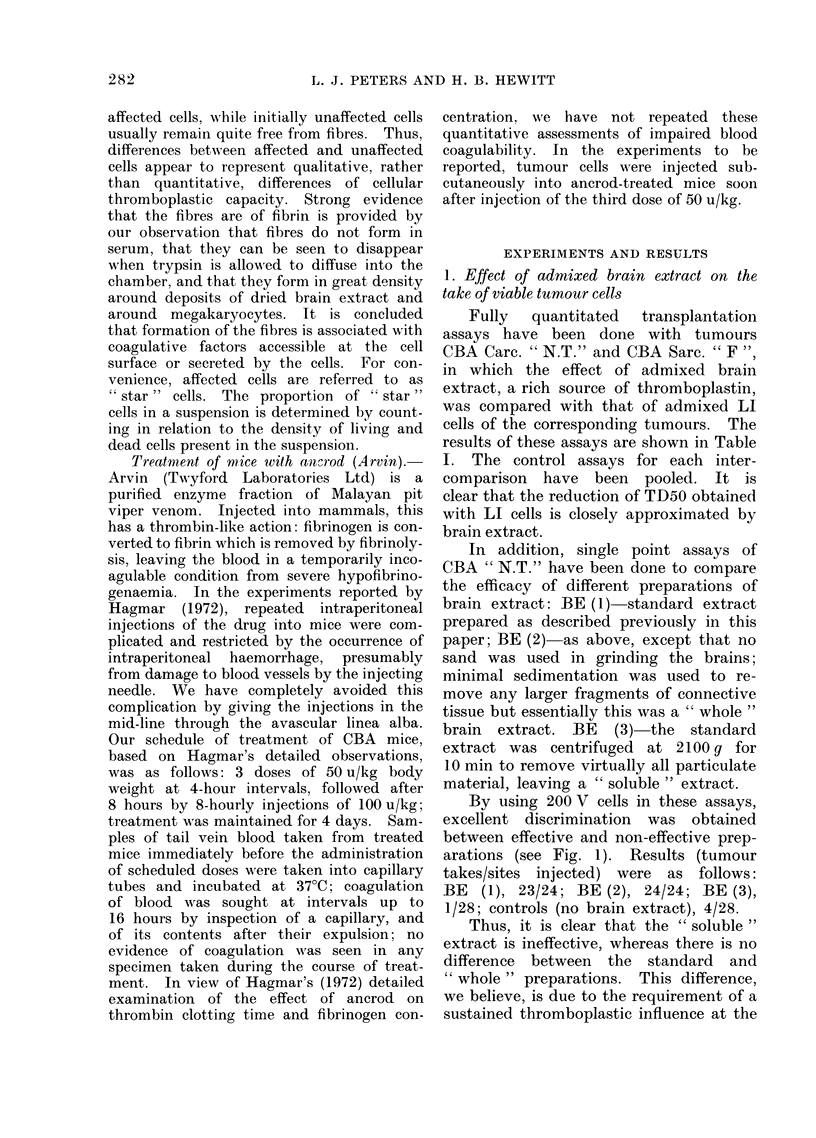

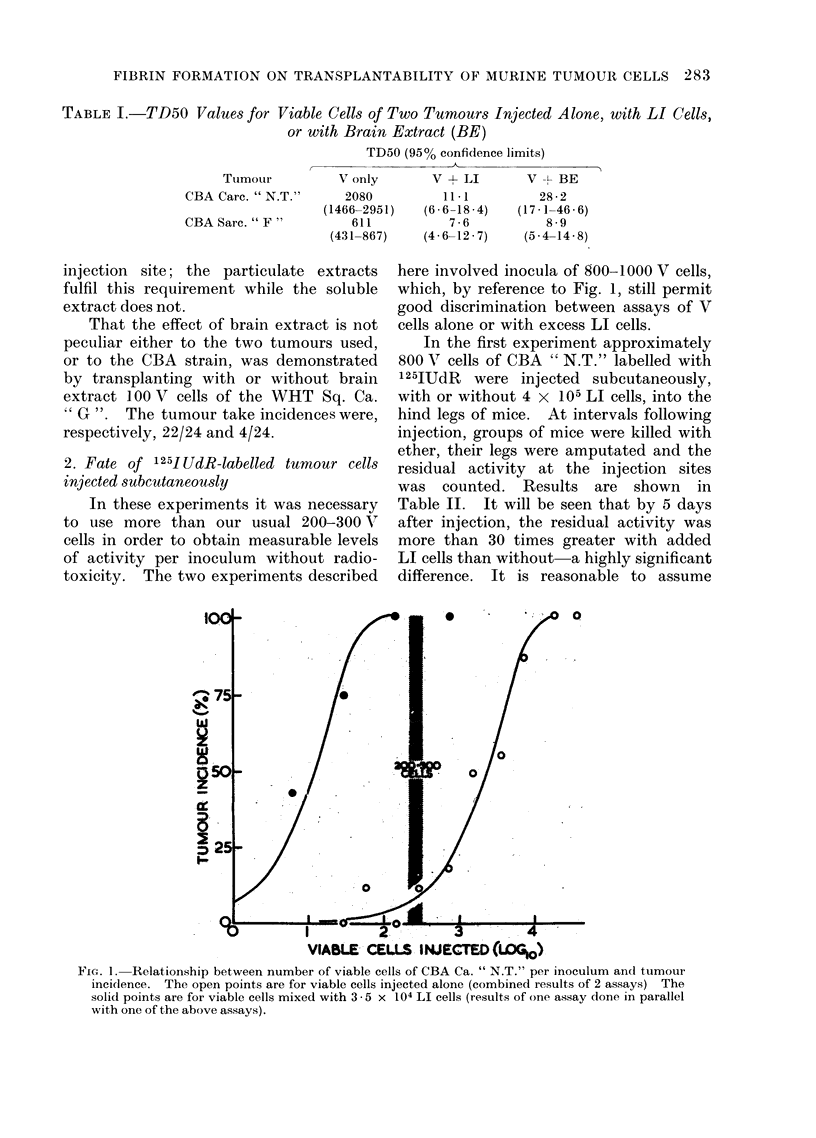

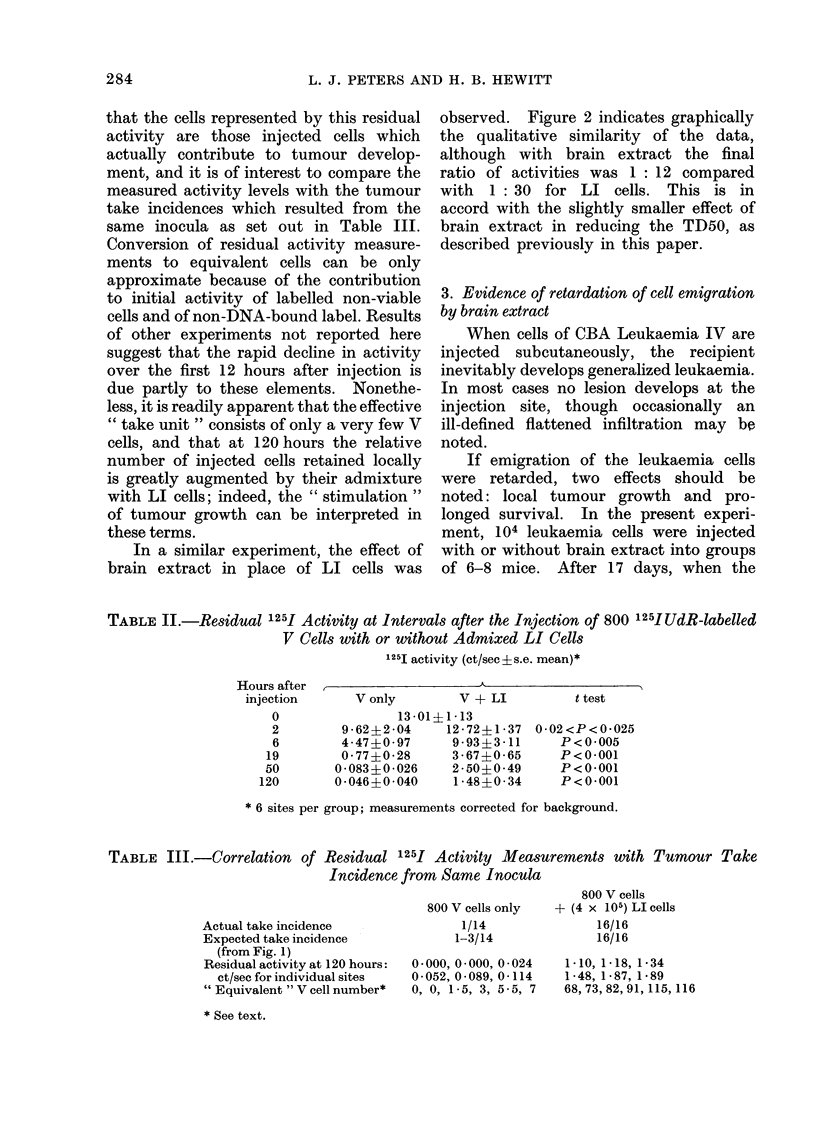

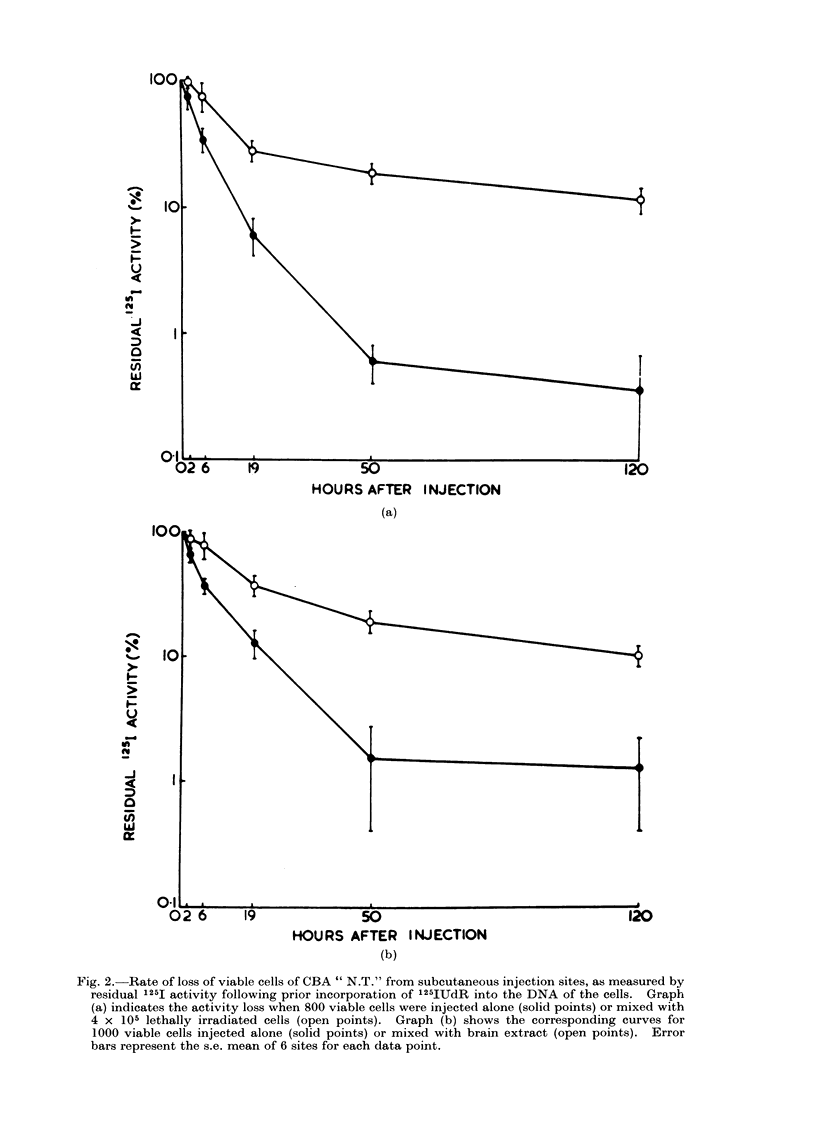

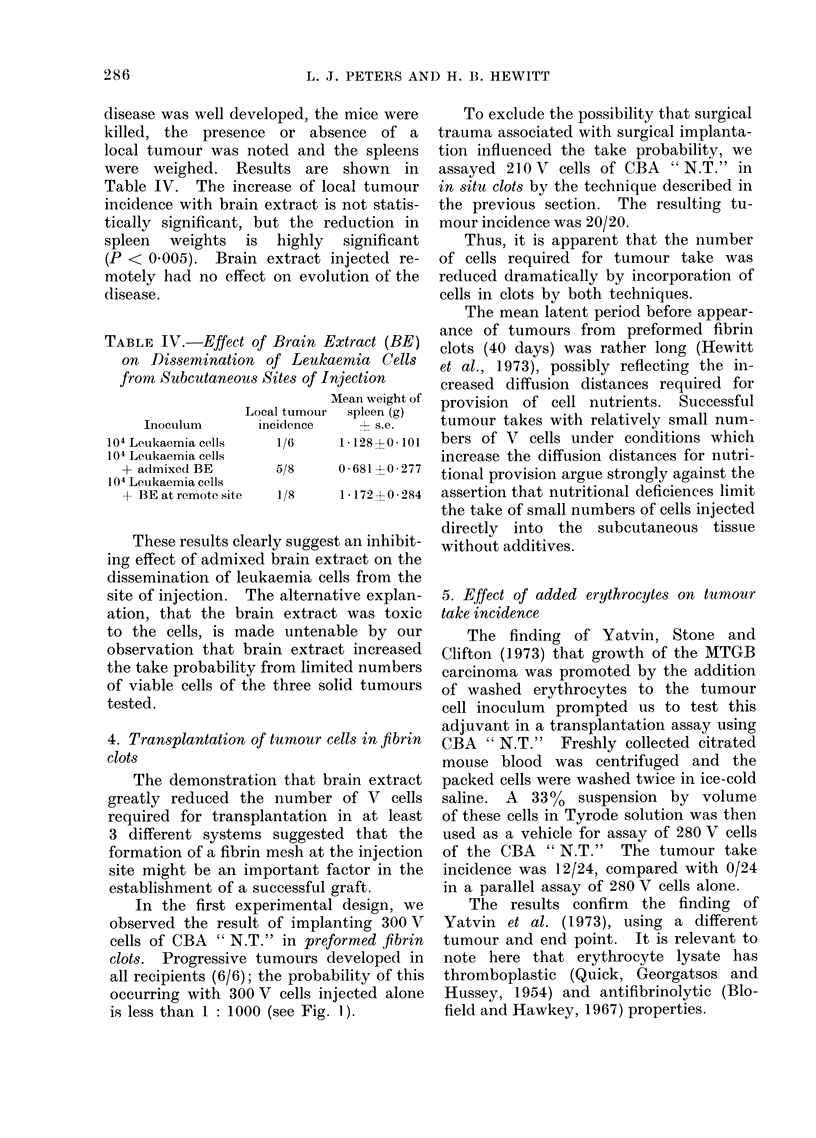

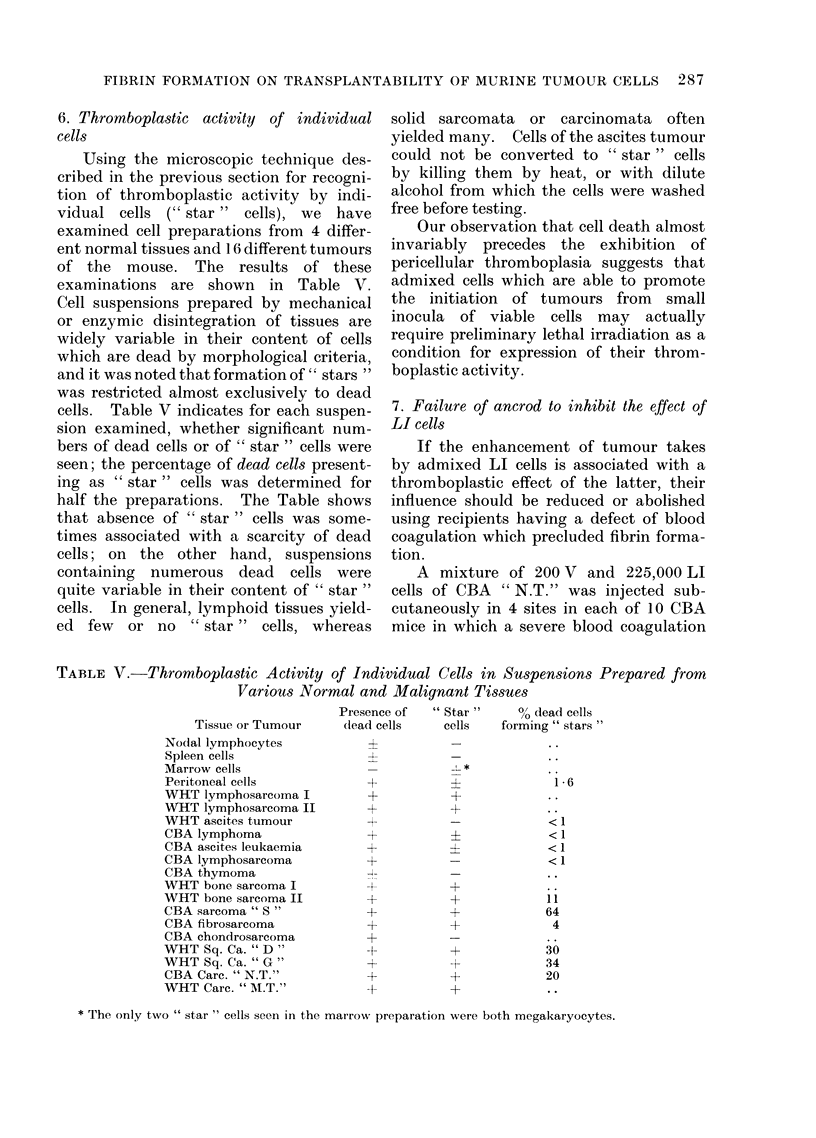

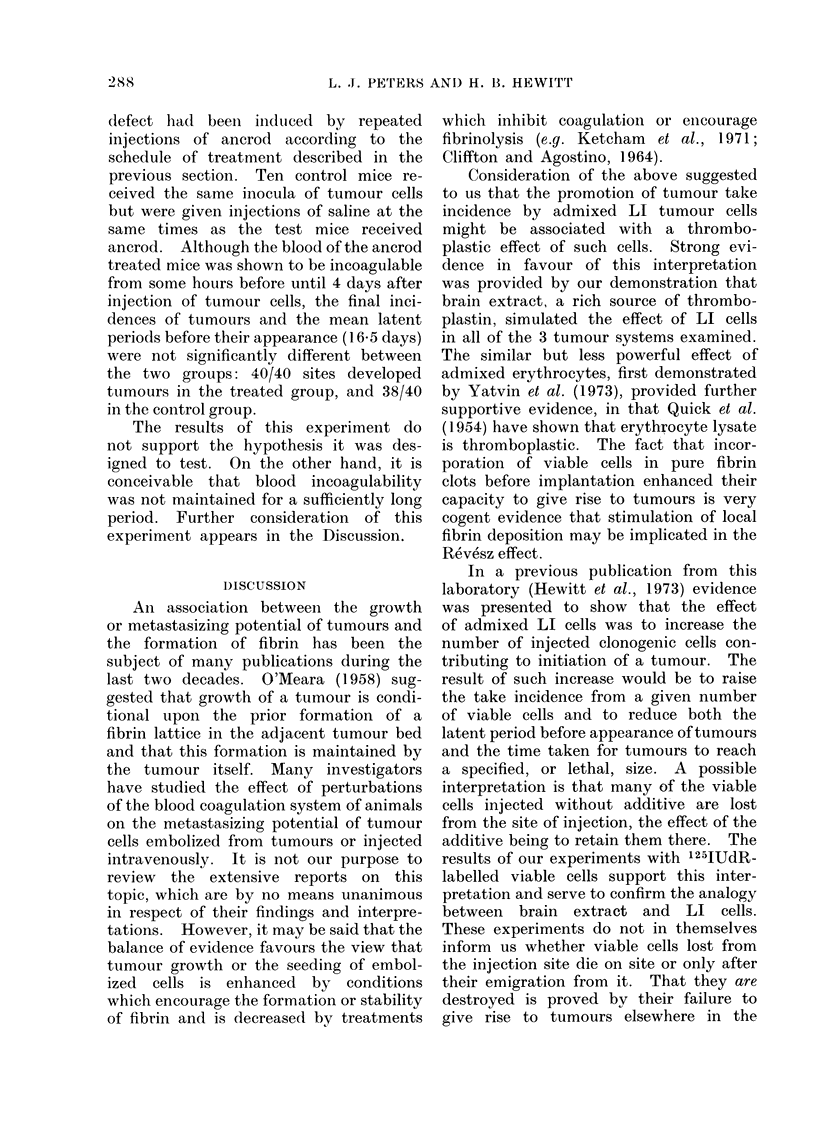

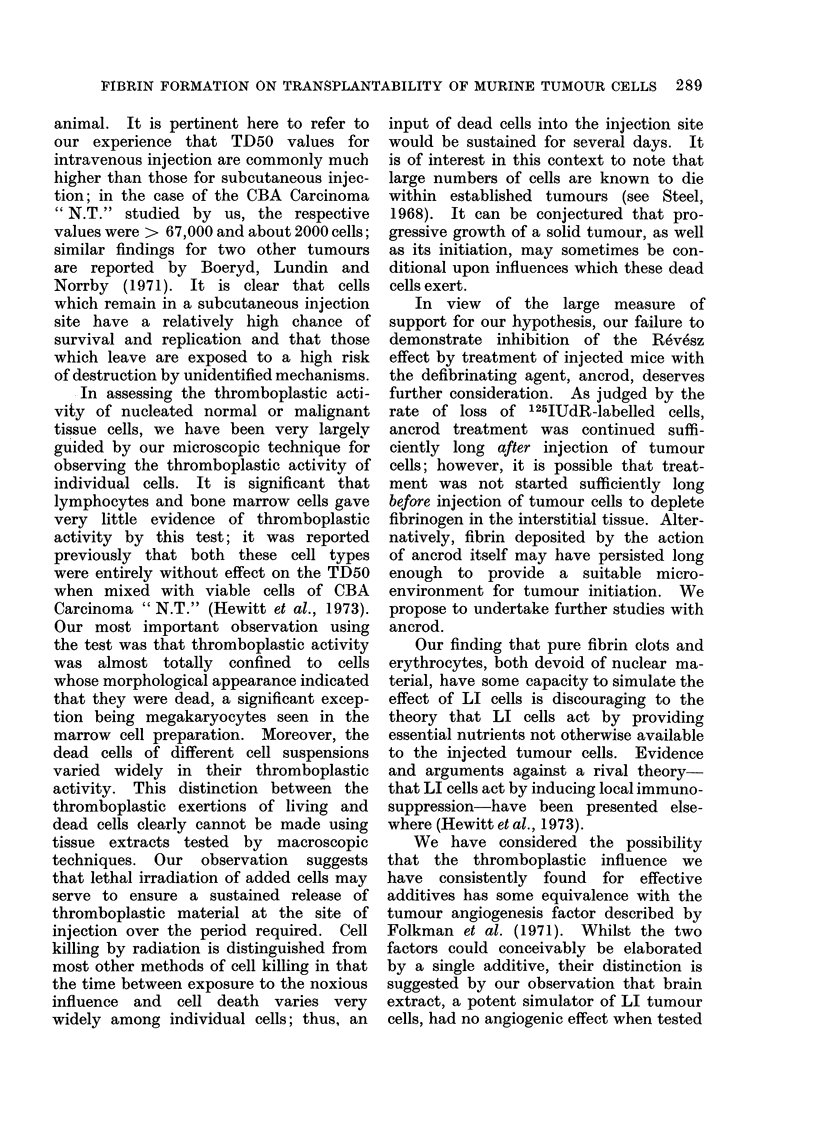

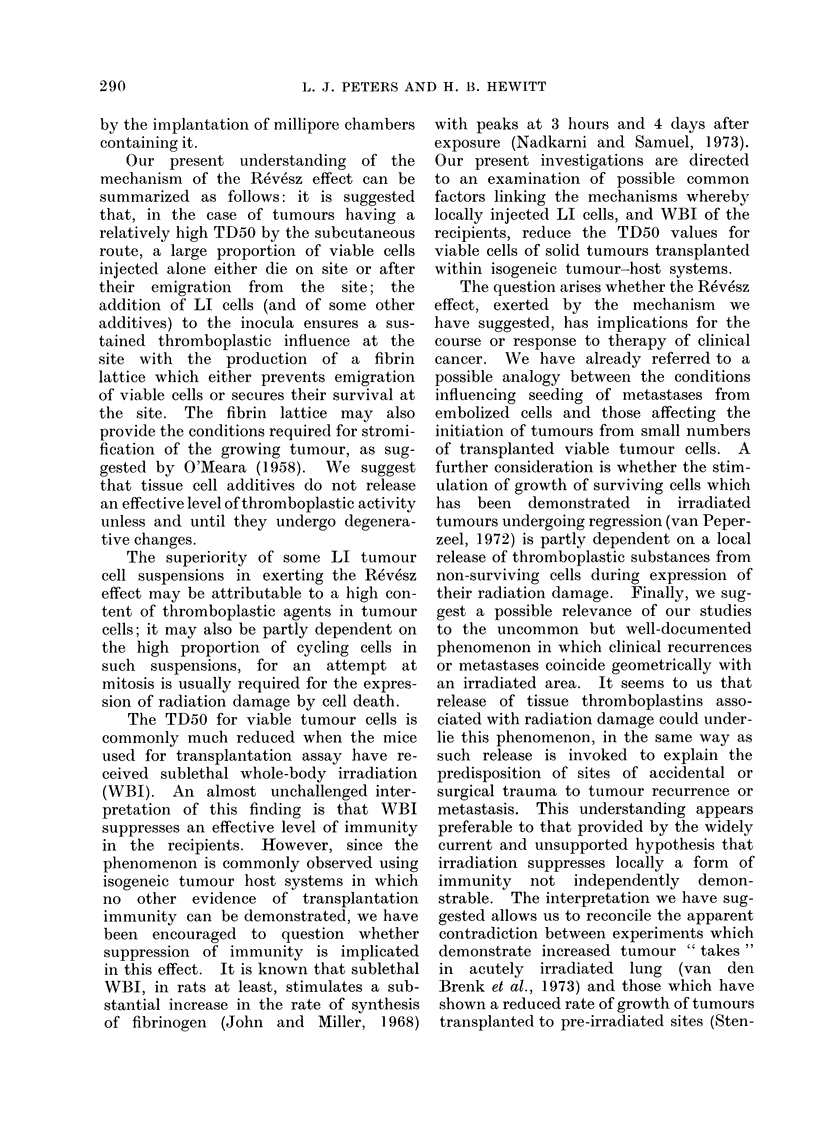

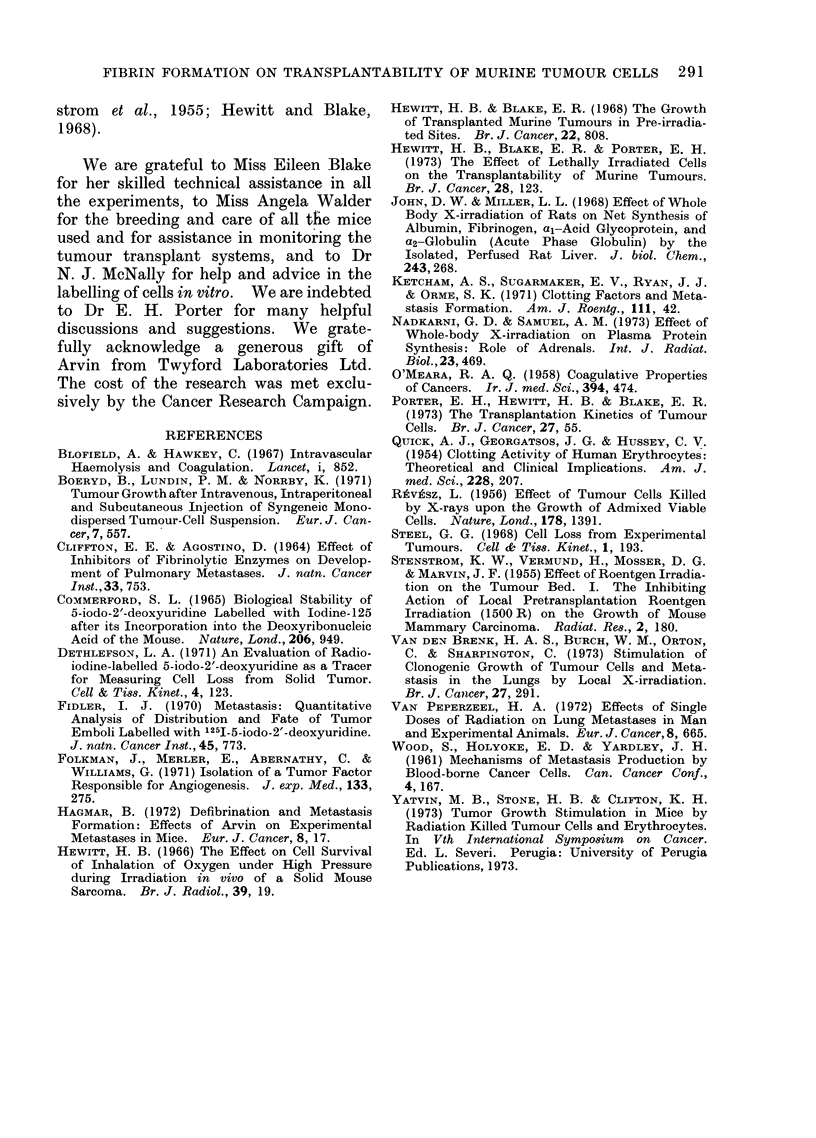


## References

[OCR_01316] Boeryd B., Lundin P. M., Norrby K. (1971). Tumour growth after intravenous, intraperitoneal and subcutaneous injection of syngeneic monodispersed tumour-cell suspension.. Eur J Cancer.

[OCR_01323] CLIFFTON E. E., AGOSTINO D. (1964). EFFECT OF INHIBITORS OF FIBRINOLYTIC ENZYMES ON DEVELOPMENT OF PULMONARY METASTASES.. J Natl Cancer Inst.

[OCR_01329] Commerford S. L. (1965). Biological stability of 5-iodo-2'-deoxyuridine labelled with iodine-125 after its incorporation into the deoxyribonucleic acid of the mouse.. Nature.

[OCR_01341] Fidler I. J. (1970). Metastasis: quantitative analysis of distribution and fate of tumor emboli labeled with 125 I-5-iodo-2'-deoxyuridine.. J Natl Cancer Inst.

[OCR_01347] Folkman J., Merler E., Abernathy C., Williams G. (1971). Isolation of a tumor factor responsible for angiogenesis.. J Exp Med.

[OCR_01353] Hagmar B. (1972). Defibrination and metastasis formation: effects of arvin on experimental metastases in mice.. Eur J Cancer.

[OCR_01364] Hewitt H. B., Blake E. R. (1968). The growth of transplanted murine tumours in pre-irradiated sites.. Br J Cancer.

[OCR_01369] Hewitt H. B., Blake E., Proter E. H. (1973). The effect of lethally irradiated cells on the transplantability of murine tumours.. Br J Cancer.

[OCR_01358] Hewitt H. B. (1966). The effect on cell survival of inhalation of oxygen under high pressure during irradiation in vivo of a solid mouse sarcoma.. Br J Radiol.

[OCR_01375] John D. W., Miller L. L. (1968). Effect of whole x-irradiation of rats on net synthesis of albumin, fibrinogen, alpha-1-acid glycoprotein, and alpha-2-globulin (acute phase globulin) by the isolated, perfused rat liver.. J Biol Chem.

[OCR_01383] Ketcham A. S., Sugarbaker E. V., Ryan J. J., Orme S. K. (1971). Clotting factors and metastasis formation.. Am J Roentgenol Radium Ther Nucl Med.

[OCR_01312] Lewis R. A. (1967). Sickle-cell anaemia in G.-6-P.D. deficiency.. Lancet.

[OCR_01388] Nadkarni G. S., Samuel A. M. (1973). Effect of whole-body x-irradiation on plasma-protein synthesis: role of adrenals.. Int J Radiat Biol Relat Stud Phys Chem Med.

[OCR_01394] O'MEARA R. A. (1958). Coagulative properties of cancers.. Ir J Med Sci.

[OCR_01398] Porter E. H., Hewitt H. B., Blake E. R. (1973). The transplantation kinetics of tumour cells.. Br J Cancer.

[OCR_01403] QUICK A. J., GEORGATSOS J. G., HUSSEY C. V. (1954). The clotting activity of human erythrocytes: theoretical and clinical implications.. Am J Med Sci.

[OCR_01409] REVESZ L. (1956). Effect of tumour cells killed by x-rays upon the growth of admixed viable cells.. Nature.

[OCR_01418] STENSTROM K. W., VERMUND H., MOSSER D. G., MARVIN J. F. (1955). Effects of roentgen irradiation on the tumor bed. I. The inhibiting action of local pretransplantation roentgen irradiation (1500 r alpha) on the growth of mouse mammary carcinoma.. Radiat Res.

[OCR_01426] Van Den Brenk H. A., Burch W. M., Orton C., Sharpington C. (1973). Stimulation of clonogenic growth of tumour cells and metastases in the lungs by local x-radiation.. Br J Cancer.

[OCR_01433] van Peperzeel H. A. (1972). Effects of single doses of radiation on lung metastases in man and experimental animals.. Eur J Cancer.

